# Type-A Gelatin-Based Hydrogel Infiltration and Degradation in Titanium Foams as a Potential Method for Localised Drug Delivery

**DOI:** 10.3390/polym15020275

**Published:** 2023-01-05

**Authors:** Hanaa Mehdi-Sefiani, Víctor Perez-Puyana, Francisco José Ostos, Ranier Sepúlveda, Alberto Romero, Mohammed Rafii-El-Idrissi Benhnia, Ernesto Chicardi

**Affiliations:** 1 Department of Engineering and Materials Science and Transportation, University of Seville, 41012 Seville, Spain; 2 Department of Chemical Engineering, Faculty of Chemistry, University of Seville, 41012 Seville, Spain; 3 Clinical Unit of Infectious Diseases, Microbiology and Parasitology, Institute of Biomedicine of Seville (IBiS), Virgen del Rocío University Hospital, CSIC, University of Seville, 41012 Seville, Spain; 4 Department of Medical Biochemistry, Molecular Biology, and Immunology, School of Medicine, University of Seville, 41012 Seville, Spain

**Keywords:** gelatin, hydrogel, solid foams, porous titanium, infiltration, degradation rate

## Abstract

A gelatin-based hydrogel was infiltrated and degraded-released in two different titanium foams with porosities of 30 and 60 vol.% (Ti30 and Ti60 foams) and fabricated by the space holder technique to evaluate its potential to act as an innovative, alternative, and localised method to introduce both active pharmaceutical ingredients, such as antibiotics and non-steroidal anti-inflammatory drugs, and growth factors, such as morphogens, required after bone-tissue replacement surgeries. In addition, the kinetic behaviour was studied for both infiltration and degradation-release processes. A higher infiltration rate was observed in the Ti60 foam. The maximum infiltration hydrogel was achieved for the Ti30 and Ti60 foams after 120 min and 75 min, respectively. Further, both processes followed a Lucas-Washburn theoretical behaviour, typical for the infiltration of a fluid by capillarity in porous channels. Regarding the subsequent degradation-release process, both systems showed similar exponential degradation performance, with the full release from Ti60 foam (80 min), versus 45 min for Ti30, due to the greater interconnected porosity open to the surface of the Ti60 foam in comparison with the Ti30 foam. In addition, the optimal biocompatibility of the hydrogel was confirmed, with the total absence of cytotoxicity and the promotion of cell growth in the fibroblast cells evaluated.

## 1. Introduction

All materials employed in the manufacture of orthopaedic implants must comply with the major characteristics related to stress-loading and long-term clinical success, such as chemical inertia, high strength under monotonic and cyclic loads (fatigue strength), low elastic modulus to prevent stress shielding [1], high corrosion resistance under body fluids [2], ease in shaping and non-toxicity, osseointegration, and excellent biocompatibility [3]. Specifically, for osseointegration, a great influence is exerted by the chemical nature, the topography, and the roughness of the surface [4]. In turn, for the biocompatibility, the size, shape, and composition of the materials, together with the chemical stability, roughness, and wettability of the surfaces, are the main factors [5,6].

The materials most widely utilised to fabricate the orthopaedic implants for bone replacements include: surgical-grade stainless steel 316 L; pure titanium (cpTi); certain Ti alloys, such as Ti6Al4V and Ti6Al7Nb (recently developed to replace the Ti6Al4V due to the toxicity of V); and Roxolid^®^ (83–87%Ti-13–17%Zr) [7,8]. The types of stainless steel are used for temporary orthopaedic implants as internal fixation devices [9], while for permanent orthopaedic implants, cpTi and Ti6Al4V are the most commonly employed [10].

Regarding cpTi and Ti6Al4V, commercially pure Ti is referred to as a non-cytotoxic material [11], while Ti6Al4V shows cytotoxicity due to the presence of V [12]. In addition, although their osseointegration and biocompatibility are appropriate, both still need to be improved. The implant failures are often related to the bioinert nature of the Ti which hinders its rapid osseointegration into the surrounding bone tissue and delays the implant attachment, causing premature failure due to micromovements [13]. Furthermore, surgical traumas can produce inflammation and, consequently, a lack of osseointegration [14]. The inflammation healing response involves the adhesion of serum proteins to the implant, which promotes bacterial attachment to the biomaterial surface, thereby producing hyperplastic soft tissues, suppuration, colour changes of the marginal peri-implant tissues, and gradual bone loss [15].

Furthermore, the functionalization and modification of the implant surface are the strategies applied to enhance the osseointegration of the implants. For example, an increase of the implant roughness [16] is applied, as is a modification of the chemical nature of the surface (deposition of a protein or peptide coating [17], while anodizing to increase the bioactive oxide layers [13]), the modification of the morphology characteristics (presence of nano, micro, or macrostructures), and the presence of residual stress, impurities, etc. [18].

On the other hand, all biomaterials are recognised as foreign materials for the human body and cause an initial host response, usually in the form of a short-term inflammatory process together with bacteria and infectious processes, but also a long-term implant rejection [19]. In general, although Ti and Ti6Al4V show excellent biocompatibility due to the formation of the protective oxide layer, this layer is sometimes damaged by wear, and the ions of the metals are then released and interchanged with the tissues or body fluids [20]. Consequently, this release can cause inflammatory processes and negative effects in the body, particularly due to the presence of V [21].

In order to counteract these short- and long-term deficiencies in biocompatibility, antibiotics (such as vancomycin and tobramycin) and/or non-steroidal anti-inflammatory drugs (NSAID), such as indomethacin, ibuprofen, ketorolac, and celecoxib, and growth factors and morphogens (recombinant human osteogenic protein-1 (rhOP-1), bone morphogenetic protein 2, rhBMP-2), are orally or parenterally supplied. However, these types of administrations can cause further health problems due to their generalised effects across the entire human body [22].

A new way to improve the osseointegration and the biocompatibility is proposed herein: the infiltration of a biodegradable and biocompatible hydrogel material [23] into the porous implants to act as a localised and controlled means of transport for drug delivery. Although the controlled release of therapeutic agents has been studied in the last few years from different materials, such as nanoparticles for intravenous administration [24], electrospun nanofibers [25], or hydrogels for localized delivery [26], it has not been specifically approached for other different biomedical applications, such as metallic implants. The hydrogels have gained special attention for drug delivery due to their low toxicity [27]. Thus, the inclusion of hydrogels in metallic implants can improve implant success by avoiding inflammation and, therefore, implant rejection. Regarding the inflammation pathways produced by implants, the main function of mitochondria is to maintain intracellular redox homeostasis in normal conditions. The mitochondria are one of the major sources of intracellular reactive oxygen species (ROS) and mitochondria-produced ROS (mtROS) and play a key role in inflammation processes. It is also worth mentioning that nuclear factor-κB (NF-κB) also exists in mitochondria [28] and is a crucial regulator of the immune response, inflammation, cell growth, and survival. In particular, the suppression of the NF-κB pathway is considered a promising therapeutic approach for regenerative medicine. In these senses, numerous systems based on hydrogels have been successfully developed for targeting mtROS and inhibiting of NF-κB activity for regenerative medicine [29,30,31]. In addition, the abovementioned drugs (antibiotics, NSADs, growth factors, and morphogens) can be included directly in the hydrogel. Moreover, the biocompatible nature of the hydrogel can increase the bioactivity of the implant surface and, consequently, can also increase osseointegration.

A hydrogel is a hydrophilic network that absorbs up to 1000 times its dry weight in water. It can be chemically stable or degradable until its dissolution [32]. Regarding its biological behaviour, a hydrogel can minimise possible mechanical irritation between the implant and the surrounding tissue due to its ability to release stress thanks to its mechanical elasticity, and it can enhance the exchange of oxygen and nutrients with human fluids because of its high permeability. The use of polymers as raw materials is highly recommended [33]. Therefore, the use of biopolymers, such as proteins, as raw materials in the biomedical field is highly recommended due to their biocompatibility, biodegradability, and stability up to 37 °C, which mimics the temperature of the human body. As a result of these properties, hydrogels are useful for numerous applications [34], such as as carriers for the release of drugs, enzymes, or growth factors [35,36]. Nevertheless, it is important to develop a suitable technique to encapsulate the drug molecules on the materials to favour an adequate release [37]. For this purpose, the hydrogels must be progressively degraded, thereby allowing the release of the factor introduced therein. Among the factors that can influence the degradation are the temperature, the raw material used for its manufacture, and the concentration used [38].

Therefore, to study the abovementioned suitability of a hydrogel as a vehicular means of localised drug transport in titanium implants, a type-A gelatin-based hydrogel was developed and was microstructurally, rheologically, and biologically characterized. This type-A gelatin protein was selected for its great biocompatibility and ability to form hydrogels [39]. Subsequently, its infiltration and degradation behaviour were evaluated in titanium foam specimens with different porosities that had been manufactured via the space-holder technique. The main novelty of this work is the combination of the fabrication and rheological and microstructural characterization of hydrogels with their infiltration and release in Ti foams previously characterized.

## 2. Materials and Methods

### 2.1. Synthesis and Characterization of the Type-A Gelatin-Based Hydrogel

A type-A gelatin (Bloom 300) with a protein content higher than 85 wt.% was used as the raw material for the development of various hydrogels. It was purchased from Sigma Aldrich S.A. (Darmstadt, Germany). Acetic acid 0.05 M (pH 3.2), supplied by Panreac Química S.A. (Barcelona, Spain), was used as the solvent.

The formation of gelatin-based hydrogels was carried out following the same protocol previously defined by Perez-Puyana et al. [40] Briefly, polymeric solutions were prepared at 1, 2, and 3 wt.% (named 1, 2, and 3%, respectively) and centrifuged at 12,000 rpm for 7 min, while maintaining the temperature of the solution at 4 °C. The gelation process was then carried out by keeping the solutions in a fridge at 4 °C for 2 h.

Subsequently, the rheological characterization of the developed gelatin-based hydrogels was carried out to evaluate their physical and mechanical properties. The strain sweep tests were performed to evaluate the linear viscoelastic range (LVR) and the critical strain (the last strain value in the linear viscoelastic region). These tests were carried out between 0.1 and 100% of strain at a constant frequency of 1 Hz and at 4 °C. Furthermore, frequency sweep tests between 0.02 and 10 Hz were also performed at constant strain (within the LVR). The elastic modulus (G′) and loss tangent (tan δ = G″/G′) at 1 Hz (G′_1_ and tan δ_1_, respectively) were analysed to improve the comparison between the systems. Moreover, flow curves between 0.02 and 200 s^−1^ were carried out to evaluate the evolution of the viscosity with the shear rate. The viscosity values at 100 s^−1^ (η_100_) were selected for the comparison of the systems. All the measurements were performed in a controlled-stress rheometer AR2000 (TA Instruments, New Castle, DE, USA) using a plate-to-plate serrated aluminium geometry of 40 mm in diameter. The rheological tests were carried out in triplicate (n = 3).

The analysis of the degradation of hydrogels was carried out by modifying a protocol previously described by Essawy et al. [41]. In addition, the gelatin-based hydrogels were prepared with 5 wt.% (with respect to the protein content) of zinc sulphate (ZnSO_4_) in their initial formulation (Sigma Aldrich, Darmstadt, Germany). Further, once formed, they were immersed in MiliQ water, and the conductivity of the solution was measured over time with a conductivity meter, the EC-Meter BASIC 30 model from Crison Instruments, Spain. The degradation of the hydrogels was related to the liberation of zinc sulphate into the medium and the subsequent increase in conductivity until a plateau was reached.

The degradation behaviour of the gelatin hydrogels was modelled following the equation defined by Korsmeyer et al. [42] for polymeric systems (Equation (1)):

(1) $$L=k\cdot t^{n}$$

where *L* is the degradation of the system and *t* is the elapsed time. The parameters obtained from this equation enable the degradation mechanism that was taking place (*n*) to be ascertained, as well as its kinetics (*k*).

The microstructural characterization was conducted using a Zeiss EVO scanning electron microscope. A pre-treatment of the samples was carried out before their observation under the microscope, which consisted of fixing the system with glutaraldehyde and osmium, followed by chemical drying based on acetone solutions. After the samples were stabilised, they were coated with a thin layer of Au/Pd to improve their conductivity, and their visualisation in the microscope was then carried out at 10 kV using secondary electrons. From the microscopy examination, the mean pore size of the hydrogels was obtained.

Finally, the cytotoxicity of the three hydrogels (1 wt.%, 2 wt.%, and 3 wt.% of gelatin) was estimated in vitro using the CyQUANT™ LDH cytotoxicity assay [43]. Several cell lines from ATCC^®^ (Manassas, Virginia, USA) were utilised: Vero E6 (normal monkey kidney epithelial cells), HeLa (human cervical carcinoma epithelial cells), U937 (human leukaemia monocytic cells), THP-1 (human leukaemia monocytic cells), and Jurkat, Clone E6-1 (human T leukaemia cells). Each cell line was seeded at 10 × 10^4^ cells/well into Nunc flat-bottomed 96-well plates (ThermoFisher Scientific) using complete D-10 or R-10 Dulbecco’s modified Eagle medium (DMEM) or Roswell Park Memorial Institute medium (RPMI) supplemented with 10% foetal bovine serum (FBS) and penicillin, incubated at 37 °C in 5% CO_2_, and used the following day (75 to 90% confluence). The FBS used in all experiments was heat-inactivated (56 °C, 30 min) prior to use to eliminate complement activity. Hydrogel solutions at various concentrations (wt.%) were added to each well, and the plates were incubated for 48 h at 37 °C in 5% CO_2_. Controls D-10 or R-10 medium alone were used as the negative control. Subsequently, 10 µL of 10X Lysis Buffer and 10 μL of sterile ultrapure water were added to each set of triplicate wells and used as the maximum lactate dehydrogenase (LDH) activity and spontaneous LDH activity, respectively. The medium from each well was later collected by means of centrifugation of the plate and was used to test the cytotoxicity of the hydrogel solutions using the CyQUANT™ LDH Cytotoxicity Assay Kit (Invitrogen™, Thermo Fisher Scientific, Waltham, MA, USA). The cytotoxicity was measured by fluorescence in a CLARIOstar^®^ (BMG LABTECH, Allmendgrün, Ortenberg, Germany). Each hydrogel concentration was measured in triplicate. The cell viability was calculated using Equation (2):

(2) $$\%\;\mathrm{Cell}\;\mathrm{viability}=100-\left(\left[\frac{\mathrm{Compound}\;-\;\mathrm{treated}\;\mathrm{LDH}\;\mathrm{activity}\;-\;\mathrm{Spontaneous}\;\mathrm{LDH}\;\mathrm{activity}}{\mathrm{Maximum}\;\mathrm{LDH}\;\mathrm{activity}\;-\;\mathrm{Spontaneous}\;\mathrm{LDH}\;\mathrm{activity}}\right]\cdot 100\right)$$

Cell viability values were also verified by the trypan blue method [44], and no significant differences were observed.

### 2.2. Fabrication and Characterization of the Titanium Foams

The two titanium foam specimens (cylinders 15 mm in height and 12 mm in diameter) with 30 vol.% and 60 vol.% of nominal porosity (known as Ti30 and Ti60, respectively) were manufactured through the space-holder technique to infiltrate with the type-A gelatin-based hydrogels. This accepted method to obtain metallic foams is based on the use of removable particles as pore formers, mixed with the metallic materials. When the space holder is removed, the volume initially occupied by the space holder is transformed into porosity. An in-depth description of this method is given by Rodriguez-Contreras, A. et al. [45].

The elemental titanium (99% purity, <325 mesh, Strem Chemicals, Newburyport, MA, USA) and sodium chloride, NaCl (99.9% purity, <35 mesh, 500 micros, Panreac Química S.L.U., Barcelona, Spain), as the space holders, were employed for the development of the titanium foam specimens. Both phases were mixed in the corresponding volumetric percentages, that is, 30 vol.% NaCl-70 vol.% Ti for the Ti30 specimen and 60 vol.% NaCl-40 vol.% Ti for the Ti60 specimen, respectively. These mixtures of powders were homogenised in a TURBULA^®^ 3D shaker mixer, model T2F (GlenMills, New Jersey, NJ, USA), at a frequency of 101 min^−1^ for 1 h. Subsequently, the mixed powders were compacted by uniaxial pressing in a universal testing machine (MALICET ET BLIN U-30, Aubervilliers, Paris, France) at 400 MPa. The green compacted cylinders obtained were immersed in hot distilled water (1 L, 60 °C) and stirred at 100 rpm to promote the dissolution of the NaCl space-holder. These developed green titanium foams were then sintered in a tubular furnace (CARBOLITE ZTF 15, Sheffield, United Kingdom) at 1200 °C for 2 h, at 10 °C·m^−1^ of heating rate, free cooling, and under a high vacuum atmosphere (10^−4^ mbar).

The fabricated specimens of sintered Ti30 and Ti60 were deeply characterized. Thus, their absolute density and the total, open, and closed porosities were calculated by the Archimedes method for porous metallic materials (ASTM B962-17). On the other hand, X-ray diffraction (XRD) was performed to elucidate the phases existing in both the Ti30 and Ti60 specimens. The diffractograms were collected on the fully-polished surfaces of the radial cross-section of the Ti foams by a PANalytical X’Pert Pro instrument (Malvern Panalytical Ltd., Malvern, UK) with Bragg-Brentano θ/θ geometry, a Cu-Kα radiation source (45 kV, 40 mA), a secondary Kb filter, and a X’Celerator detector, by scanning between 30° and 80°, with a step size of 0.03° and an 800 s·step^−1^.The Crystallography Open Database (COD) was employed to elucidate the crystalline phases.

In the determination of the morphology, pore-size distribution, and circular equivalent diameter of the pores (D_eq_), defined as the diameter of a circle with the same area as the pore, an image analysis was carried out on those fully polished surfaces of the Ti30 and Ti60 foams. Specifically, the radial cross-section images were collected with a Nikon Eclipse MA100N optical microscope. In turn, the image analysis was carried out using the Image Pro Plus^®^ software. Three images at different heights were studied for each of the Ti30 and Ti60 specimens.

Finally, a strain-stress curve for the Ti60 specimen was obtained by compression testing in a universal Instron mechanical testing machine (model no. 6025). The tests were carried out according to the ASTM E9-09 standard, with a constant strain rate of 0.05 mm/min and a maximum application load of 100 kN, the maximum value available for the machine. Thus, three cylindrical specimens with a diameter of 12 mm and a height of 15 mm (diameter/height rate equal to 0.8), as recommended for “short solid cylindrical specimens” in ASTM E9-09, were tested.

### 2.3. Infiltration-Degradation for the Gelatin-Based Hydrogels into the Ti30 and Ti60 Foams

The Ti30 and Ti60 foams were used to infiltrate, subsequently degrade, and release the 3% hydrogel into the open porosities of both specimens. In particular, the Ti30 and Ti60 specimens were submerged in the 3% hydrogel, and the infiltration process was performed at room temperature. The infiltration of the hydrogel was carried out by capillarity with the immersion of the Ti foam in the hydrogel. The penetration process was monitored by the measurement of the weight increase associated with the infiltration during the immersion time. The infiltration processes were stopped when the total open porosity was infiltrated by the hydrogel and/or a stationary stage was reached. Subsequently, this process was replicated with the 3% hydrogel synthesised in the presence of zinc sulphate, ZnSO₄ (5 wt.% with respect to the protein content in the 3% hydrogel), to study the release of the 3% hydrogel by the conductivity technique under the same aforementioned conditions.

## 3. Results

### 3.1. Synthesis and Characterization of the Gelatin-Based Hydrogels

The rheological characterization of the hydrogels started with the strain sweep tests to obtain the linear viscoelastic range and the critical strain of each system. According to the results (Table 1), as a general trend, there is an increase in the critical strain by increasing the biopolymer concentration, reaching the maximum for the 2% hydrogel. Similar results were found by Sánchez-Cid et al. [46], who demonstrated that a higher raw material concentration led to systems with improved mechanical properties.

**Figure 1:**
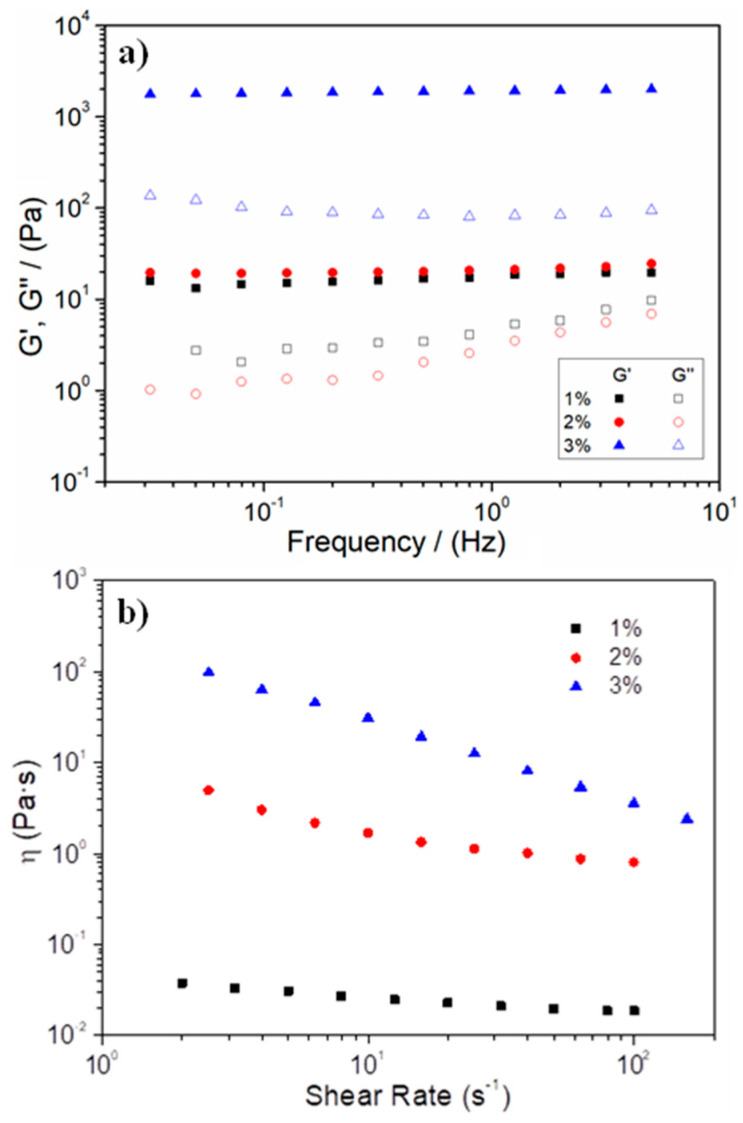
(**a**) Frequency sweep tests and (**b**) evolution of the viscosity with the shear rate for the gelatin-based hydrogels fabricated at different gelatin concentrations (1, 2, and 3 wt.%). The average profile of a triplicate (n = 3) was included.

The frequency sweep tests were also carried out, and the results obtained are shown in Figure 1a. According to the profiles obtained for the three systems, G′ values are always higher than G″ values, thus showing a predominantly elastic behavior. Furthermore, the three systems showed a constant profile in the frequency range studied, thereby corroborating their stability. In comparing the elastic modulus and loss tangent at 1 Hz included in Table 1, it can be observed how the G′ values of the systems increased with increasing concentration, thereby highlighting the 3% hydrogel. The increase in concentration led first to an increase in the critical strain and then to an increase in elastic behaviour. The differences shown in Table 1 may be due to the presence of numerous protein chains, which would lead to better interaction and structuring of the system and, therefore, a significant increase in its elastic modulus. In fact, the tan δ values shown in Table 1 evidenced how a higher concentration increased the solid character of the hydrogels since the lower the tan δ, the greater the difference between G′ and G″ values. This difference highlights the better mechanical resistance of the 3% hydrogel, which can provide benefits from its use as a biodegradable biomaterial because it can withstand greater mechanical stress during the cell-growth and proliferation stage.

On the other hand, flow curves were also carried out to analyse the viscosity of the systems and their evolution with the shear rate. As can be observed in Figure 1b, all systems presented pseudoplastic behaviour since viscosity decreases as shear rate increases, thereby rendering hydrogels more fluid. This behaviour is more evident in the system at 3%, since the hydrogels prepared at a lower concentration showed a slight decrease followed by a plateau at higher shear stresses, for which the viscosity remained constant. A comparison of the systems revealed that the higher the amount of gelatin, the higher the viscosity of the hydrogel. This higher viscosity is caused by a greater reinforcement of the hydrogel, probably due to greater structuration of the internal network [47].

Therefore, after the rheological evaluation of the hydrogels, 2% and 3% hydrogels can be presented as those with the best properties to be used as biodegradable biomaterials, due to their higher viscosity and critical strain.

**Figure 2:**
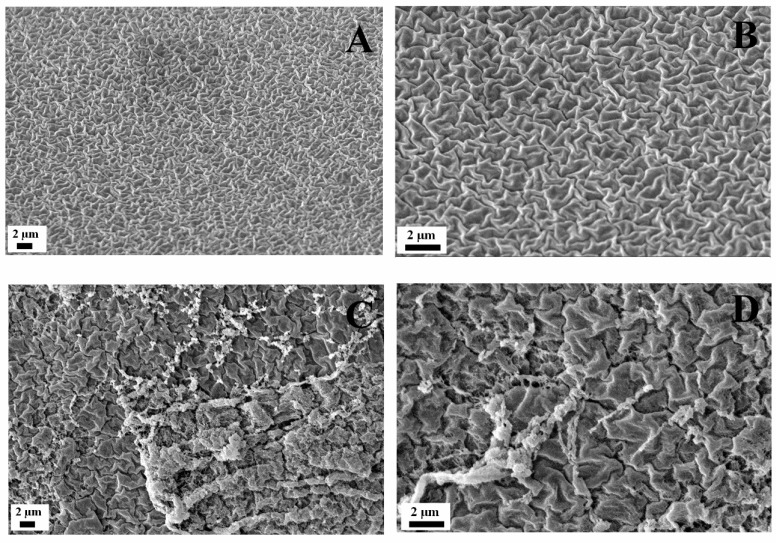
SEM images of the gelatin-based hydrogels fabricated at different concentrations: (**A**,**B**) 2 wt.%, and (**C**,**D**) 3 wt.%.

A microstructural evaluation of the gelatin-based hydrogels developed has been carried out by SEM imaging. Figure 2 shows the microstructures of the hydrogel at 2% and 3% and at 2000× and 4000×. On comparing the two systems, the 3% hydrogel presented a large structure that generated a less compact structure and, consequently, higher pore-size values. The largest long-range structure explains the greater solid character of the 3% hydrogel system determined from the rheological tests. The pore size of both systems was calculated, revealing that the 3% hydrogel presented a mean pore size ca. 3 times higher than that of the 2% (0.46 ± 0.15 and 0.16 ± 0.04 um, respectively). The SEM images obtained revealed a similar microstructure as previously shown by Sanchez-Cid et al. with the development of collagen-based hydrogels [48] or by George et al. with gelatin-based hydrogels [49].

**Figure 3:**
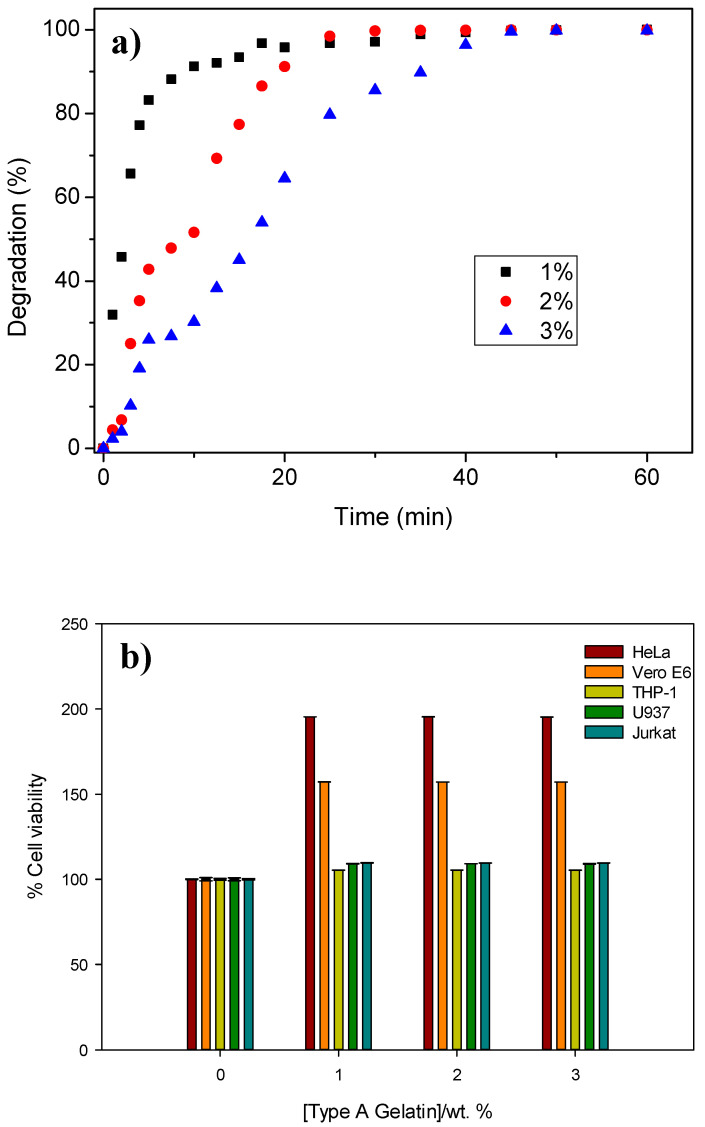
(**a**) Degradation behaviour and (**b**) cell viability values for the gelatin-based hydrogels fabricated at different concentrations. The average values of a triplicate (n = 3) were included.

In addition to the rheological behaviour of the hydrogels, their degradation was also analyzed. Figure 3a shows the degradation profiles of the different hydrogels as a function of the gelatin concentration. According to the results obtained, the hydrogels with a higher protein concentration presented a slow liberation and, therefore, required a longer time to be completely degraded, specifically 30 min for the 1% and 2% hydrogels and 45 min for the 3% hydrogel.

The results for the fitting of the degradation curves are also summarised in Table 1. The 1% hydrogel had a greater kinetic constant (k) than the 3% hydrogel, and hence the hydrogel prepared with a lower protein concentration showed a faster release, thereby correlating with the assertion observed in Figure 3a. In addition, according to the values of n defined by Korsmeyer et al. [42], 1% of the hydrogels did not have a specific release mechanism, 2% presented a diffusion release process, and the degradation of the remaining 3% was mainly due to the relaxation of protein chains.

In order to investigate the cytotoxicity of the gelatin-based hydrogel, an LDH assay was used. Figure 3b shows the results obtained in different cell lines after 48 h. The gelatin-based hydrogels do not show any cytotoxicity in the different hydrogel concentrations employed, showing a different behaviour than the studies Ahmadian et al. conducted that revealed that the viability of biopolymer-based systems decreased with concentration [50]. It should be borne in mind that the hydrogel promotes cell growth in the fibroblast cells evaluated due to the presence of amino acids with numerous biological functions in their structure [51]. Therefore, these results are in agreement with those found by other authors [51,52,53,54].

According to the characterization of the different hydrogels fabricated, the system at 3% presented the most suitable properties for the infiltration of Ti foams, due to its greater solid character and the larger pore sizes found in its network.

### 3.2. Fabrication and Characterization of the Titanium Foams

**Figure 4:**
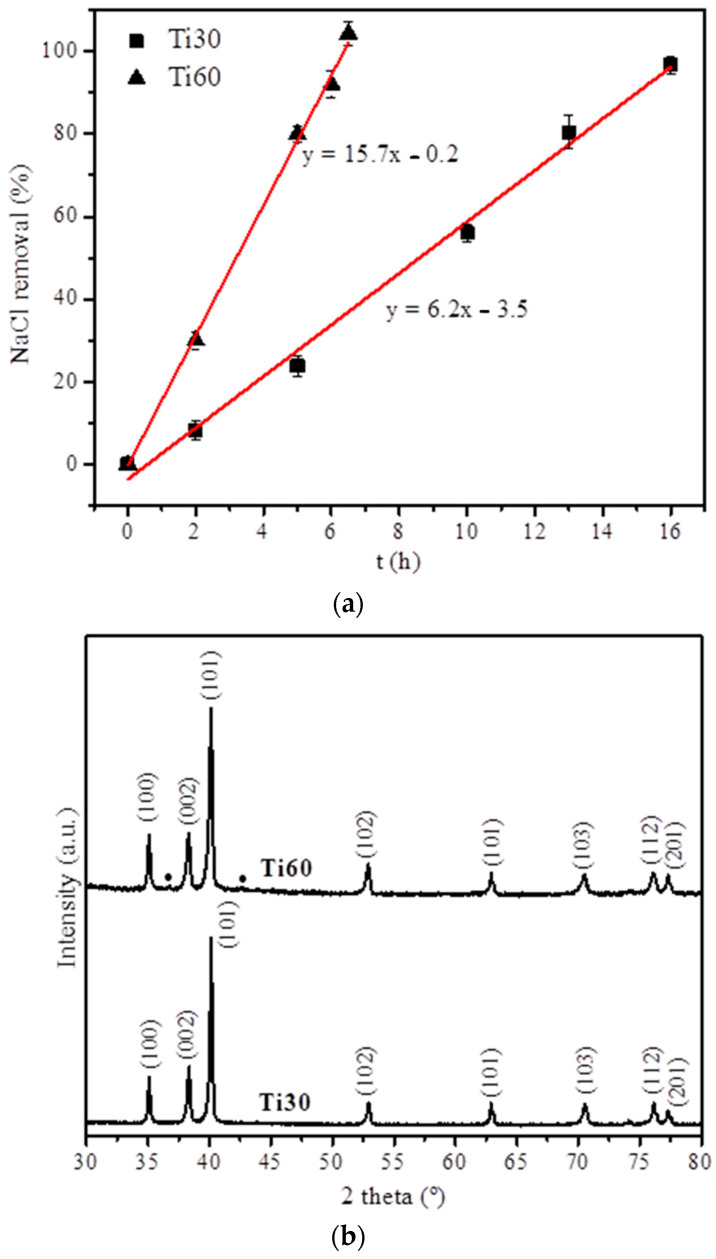
(**a**) Evolution of the NaCl removal with the immersion time for the set of Ti30 and Ti60 specimens. Continuous lines showed the linear fitting for these NaCl removal processes. The fitting equations of the linear fitting are displayed on the right-hand side of each line. (**b**) The corresponding X-ray diffraction patterns for both Ti30 and Ti60 specimens. Between brackets are the crystallographic planes for the hexagonal Ti (P63/mmc) found in both specimens. (●) Unindexed, slight peaks were observed in the Ti60 specimens, probably corresponding to titanium oxide (TiO_2_).

In the manufacture of the Ti30 and Ti60 foams, the first monitored step involved the evolution of NaCl removal (Figure 4a). Specifically, the maximum NaCl elimination, determined by the weight loss of the foams, was reached after 16 h and 6.5 h for the Ti30 and Ti60 foams, respectively. Regarding the slope of the lineal fitting, the removal rate was approximately 6.2 wt.% and 15.7 wt.% of the total NaCl per hour. Additionally, for the Ti60 foam, a slightly higher NaCl removal percentage than 100% was determined: 104%. This can be explained by the small amount of Ti that comes off the surface of the green body of the Ti60 specimen due to the handling during the sample extraction and submersion in the distilled water. In contrast, for the Ti30 specimen, the maximum NaCl removal was 96.1 ± 2.1%. In this case, small amounts of NaCl can be encapsulated by Ti particles that block the penetration of the distilled water and the full NaCl dissolution.

Subsequently, after the NaCl removal and the sintering step, the absolute density, relative densification, and open and closed porosities of the Ti30 and Ti60 foams were determined by the Archimedes method (Table 2).

It is first observed that the absolute densities determined for both sets of Ti30 and Ti60 specimens are lower than the theoretical density for Ti at 20 °C, 4.5 g·cm^−3^. This aspect suggests high porosity in both cases: 61.0 ± 2.7 vol.% and 39.6 ± 0.1 vol.% for Ti30 and Ti60, respectively. In other words, the total average porosity calculated for these foams was 39.0 ± 2.7 vol.% for Ti30 and 60.4 ± 0.1 vol.% for Ti60. These values are close to the nominal percentage porosities for Ti30 and Ti60, which suggests that this powder metallurgy process is valid for the manufacture of the Ti foams. The slightly higher total porosity measured can be attributed to the inherent porosity that remained between the Ti particles after the sintering process. The higher the Ti amount for Ti30, the higher the inherent residual porosity and, consequently, the higher the deviation from the nominal porosity. On the other hand, the most important aspect is the presence of a high level of open porosity, that is, the porosity required for the subsequent 3% hydrogel infiltration and degradation-release, for which 34.3 ± 2.1 and 43.8 ± 3.3 vol.% of open porosity for Ti30 and Ti60 were determined. In turn, the closed porosity, not available for the hydrogel infiltration, was 4.7 ± 2.5 and 16.6 ± 6.3 vol.% for Ti30 and Ti60, respectively. The high level of closed porosity for Ti60 can be attributed to the closure process of the interconnected porosity initially existing in the green specimens after NaCl removal. This effect is less noticeable for Ti30 due to the initially lower interconnected porosity as a direct consequence of the lower NaCl amount. The final open porosities obtained for both the Ti30 and Ti60 sets of specimens were enough to study the influence on the infiltration and degradation processes of the gelatin-based hydrogel.

Subsequently, an XRD analysis was carried out throughout the sample height over several radial polished cross-sections for the Ti30 and Ti60 specimens. The X-ray diffraction patterns for Ti30 and Ti60 foams (Figure 4b) showed practically a single phase across the entire sample, corresponding to the hexagonal elemental titanium, P63/mmc (identity code no. 1532765 in the Crystallography Open Database, COD), which suggested a practically total dissolution of NaCl during the space-holder removal step and the absence of titanium oxide after the sintering step. In addition, the Ti60 specimen could show two slight peaks, although they could not be indexed due to their low intensity. This minor phase can be attributed to the TiO_2_ phase due to the typical passivation phenomenon for titanium.

**Figure 5:**
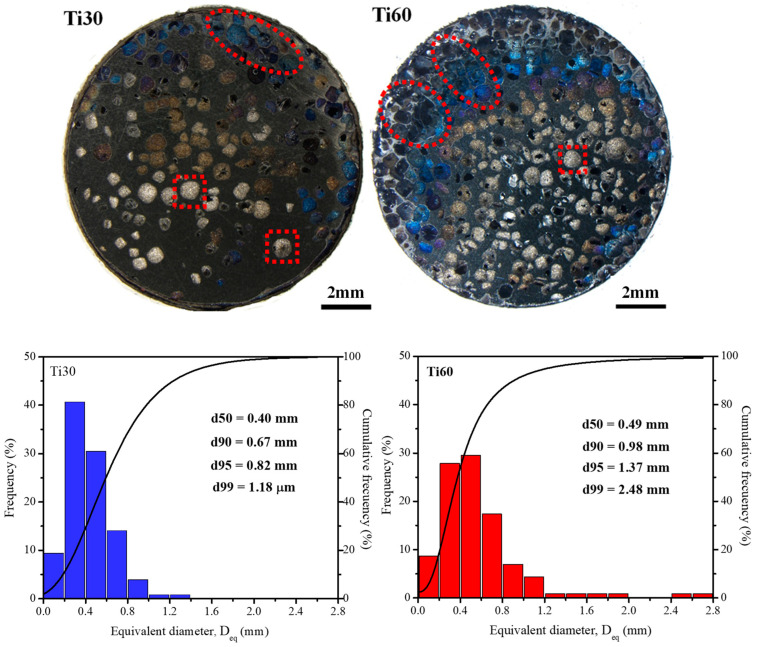
Cross-section optical micrographs (**upper** images) and pore equivalent diameter (Deq) distribution (**lower** images) for Ti30 and Ti60 foams. Dotted squares: Closed porosities; dashed ellipses: interconnected porosities.

The optical micrographs of these radial polished cross-sections are shown in Figure 5, where the higher amount of total porosity for Ti60 can be clearly observed in comparison with that of Ti30, thereby correlating the values determined by the Archimedes method. In addition, for both specimens, Ti30 and Ti60, a homogeneous distribution of pores can be observed. This porosity showed an equiaxed morphology for closed pores (marked by dotted squares in Figure 5) and elongated channel pores produced by the joining of different NaCl particles initially in contact (marked by dotted ellipses in Figure 5). Most of the elongated pores are in contact with the surface of the specimens, thereby creating the necessary open porosity for the hydrogel infiltration. This last assertion can be explained by considering the NaCl removal step. The dissolution itself and the release of NaCl from the Ti matrix were generated due to the elongated channel porosity open to the surface. It should be borne in mind that although certain internal pores appear to be closed pores, they can, in fact, have open channel porosity in a longitudinal or crosswise direction that differs from the radial cross-section of the images in Figure 5.

The pore size distribution, which combines equiaxed and elongated channel pores, was determined by image analysis and is also presented in Figure 5. The equivalent pore diameter was determined as the maximum fitting sphere. Firstly, as a general trend, the pore size distribution is wider for Ti60, which suggests a higher coalescence and interconnection of porosity in comparison with Ti30. At the same time, the percentile 50 (d50) was similar for both Ti30 and Ti60 foams, at 400 and 490 μm, respectively. These values, which are close to the initial space-holder size (500 μm), suggest that practically half of the number of pores correspond to the breaking of NaCl particles during foam fabrication. However, although this determination seems to be initially inconsistent with the lower level of closed porosity determined by the Archimedes method (see Table 2), it is necessary to highlight that the image analysis determination has been obtained from polished radial cross-sections and, obviously, the different porosities can be interconnected in other different random directions. In this respect, a small interconnect area between these sub-millimetric pores can be detected by the oils used in Archimedes, but this remains difficult to detect by image analysis.

The percentiles 95 and 99 (d95 and d99) showed higher values for Ti60 (1.37 mm and 2.48 mm) than for Ti30 (0.82 mm and 1.18 mm). Therefore, it can be assumed that the higher the percentile, the higher the equivalent diameter, and the higher the pore size. This is a clear assertion regarding the higher interconnect porosity in the Ti60 specimens in comparison with that of Ti30. Finally, most of the porosity lies within the range of 200–600 μm of the diameter equivalent. This combination of high distribution of pore size and an average pore size of 500 μm is reported by Torres-Sánchez et al. [55] to be the optimal range of porosities for the osteoblastic cellular in-growth in porous Ti implants.

**Figure 6:**
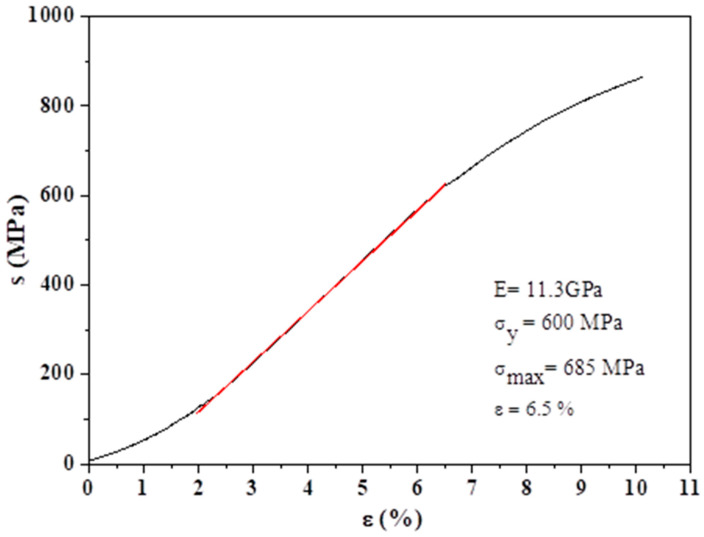
Strain-stress curve for the Ti60 specimen determined by compression testing. Inside: mechanical properties determined from the strain-stress curve and a picture of the Ti60 specimen.

Finally, the mechanical behaviour of the Ti60 specimen was carried out by compression testing (Figure 6) to determine its fit with the mechanical performance of the bone tissue. Thus, the elastic modulus determined was around 11.3 GPa, higher than trabecular bone (around 1 GPa), and close to cortical bone (15–25 GP) [56]. In addition, the determined yield strength and maximum strength reached 600 and 685 MPa, higher than the healthy cortical bone tissue (200 MPa) measured in the same compression conditions [57]. In addition, the elastic strain (ε) measured for the Ti60 specimen was around 8%, higher than the 1% for cortical bone, but already similar (2.5%) at the maximum stress supported for the cortical bone tissue. Thus, this mechanical behaviour seems adequate to be employed with this material and bone replacement tissue in terms of mechanical performance. Finally, it is important to know at this moment that the presence of the hydrogel does not affect the mechanical behaviour of the Ti60 materials and vice versa, since the critical stress for the 3% hydrogel is 0.50 ± 0.14 MPa, three orders of magnitude lower than the Ti60 specimen. In addition, the critical strain for the same 3% hydrogel is much lower (0.09 ± 0.03%) than the Ti60, indicating that the mechanical performance of the system Ti foam-hydrogel is mainly affected by the first one.

### 3.3. Infiltration-Degradation for the Gelatin-Based Hydrogels into the Titanium Foams

**Figure 7:**
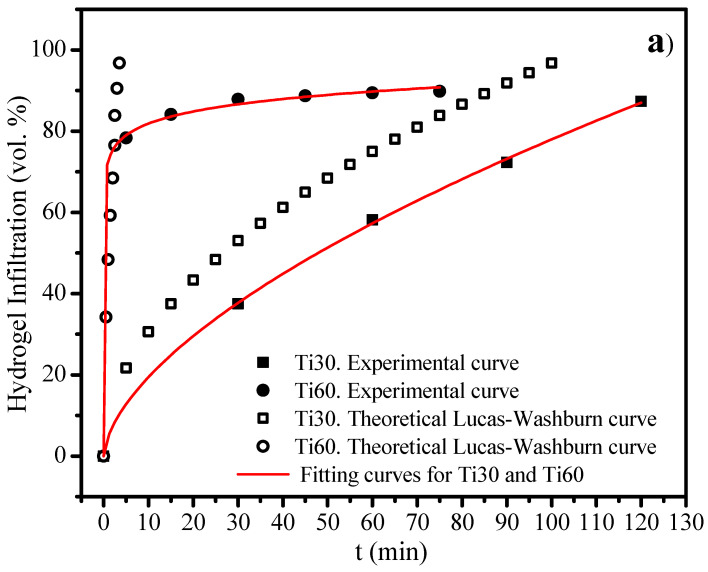

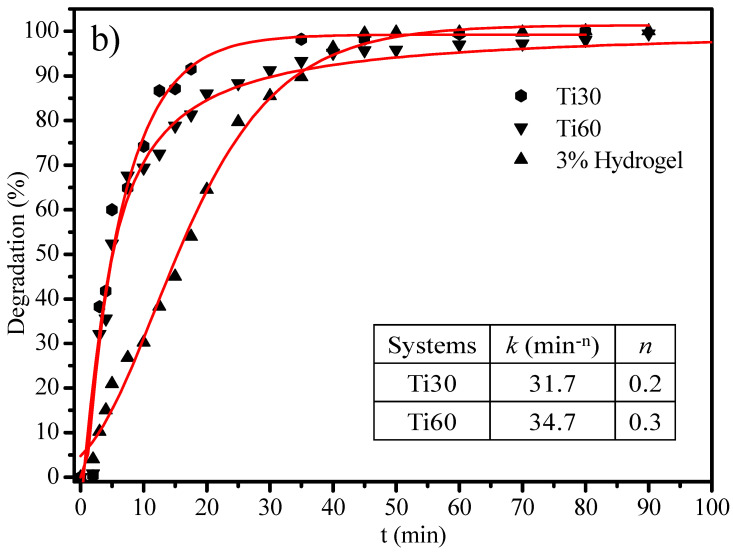
Evolution of the hydrogel infiltration (**a**) and degradation-release, (**b**) in the Ti30 and Ti60 foams. Continuous red lines marked the best fit of the infiltration processes. The degradation profile of the 3% hydrogel has also been included as a reference. Values of the kinetic constant (k) and degradation mechanism (n) obtained were also included.

Once the gelatin-based hydrogels have been characterised and the Ti30 and Ti60 foams fabricated, and the optimal 3% hydrogel has been selected in terms of its properties for biomedical applications, this hydrogel is then infiltrated into both Ti foams. The results of the infiltration process are shown in Figure 7a, where a power-law curve was observed in both Ti30 and Ti60 foams, with a generally higher infiltration rate for the 3% hydrogel in the Ti60 foam. The maximum infiltration percentage of the total interconnected porosity was therefore reached for Ti30 (88 vol.% of hydrogel 3 infiltration) after 120 min, while for the Ti60 foams, a higher value of 90.1 vol.% was reached after 75 min. In making use of the hydrogel properties determined, it was possible to simulate the theoretical curves for both infiltration processes according to the Lucas-Washburn equation [58]. These curves are also shown in hollow symbols in Figure 7a. Thus, this fitting, together with the absence of pressure or other external force and the immersion of the Ti specimens in the hydrogel, can be used to corroborate that this hydrogel is infiltrated into the narrow porous channels by capillary motion. This well-known phenomenon is based on the introduction of a fluid (hydrogel in this case) into a narrow space (Ti pores) without the assistance of any external forces, due to the intermolecular interaction between the fluid and the surrounding solid surface as a consequence of the combination of surface tension (cohesion within the liquid) and adhesive forces between the hydrogel and the Ti pore surface.

On the other hand, it can be observed that, for the Ti30 foams, the experimental and theoretical behaviours diverge with the infiltration time. However, for the Ti60 foams, both experimental and theoretical curves are similar at shorter infiltration times (up to 5 min, 80 vol.% of infiltration) and start to diverge at a later milling time. This general difference in the infiltration process for the experimental curves can be attributed to the characteristics of the interconnected porosity. While the Lucas-Washburn equation is described by a cylindrical, straight pore channel with no influence from the roughness of the surface, for the experimental Ti30 and Ti60 foams, the pore channels showed changes in radial section, tortuosity, and certain surface roughness that hinder the overall infiltration processes. Another aspect that may increase the divergence between theoretical and experimental behaviour is the presence of air trapped in the pores during the infiltration process that must diffuse from the internal interconnected porosity to the surface of the Ti foams.

Furthermore, the similar behaviour found for Ti60 foam after a short infiltration time can be explained by addressing the high number of interconnected porosities open to the specimen surface instead of a single pore as described by the Lucas-Washburn equation, which produced the absence of any hindrance to the hydrogel infiltration for Ti60 at the shortest time. This aspect is less acute for Ti30 due to the lower level of open porosity at the surface, which can be corroborated in Figure 5.

Finally, for both Ti30 and Ti60 specimens, it was not possible to reach the 100 vol.% 3% hydrogel infiltration. The presence of small-size open porosity, corroborated by the pore size equivalent distribution (Figure 5), hinders the infiltration process therein. In order to solve this handicap and reach the purpose of full hydrogel saturation of the interconnected porosity, a pressure-assisted infiltration process could be implemented.

Once the hydrogels were infiltrated into the Ti30 and Ti60 foams, their degradation was also analyzed. The results are plotted in Figure 7b. Both systems show a similar profile, with exponential growth until a plateau is reached. The modelling of the profiles reveals a similar degradation mechanism due to the similar values obtained for parameter *n*, without any predominant degradation mechanism as demonstrated by Korsmeyer et al. [42]. Furthermore, the degradation kinetics are revealed to be slightly faster for the Ti60 foam (higher k value), probably due to the greater interconnected porosity open to the surface in comparison with that of the Ti30 foam. In addition, by comparing the degradation-release of the hydrogel in the Ti30 and Ti60 specimens with the isolated hydrogel, it can be observed a little faster degradation in the last one. This aspect can be attributed to the bioinert nature of the Ti pore surface in the foams, which decreases the adhesion of the hydrogel molecules. Thus, to reduce the faster degradation of the hydrogel inside the Ti pores, the suitability of the Ti surface functionalization to increase the hydrogel’s adhesion is described. Furthermore, it should be noted that the practically total degradation of hydrogel in the three cases is not pronounced. As an example, more than 90% of degradation is reached in the three cases after 40 min, specifically, 95% of degradation for an isolated 3 wt.% hydrogel, 95% for Ti30, and 90% for Ti60.

## 4. Conclusions

In this work, a type-A gelatin-based hydrogel has been successfully infiltrated and degraded-released in two titanium foams. This process has been carried out experimentally to establish the ability of the hydrogel to be used as a vehicle for active pharmaceutical substances required after bone-tissue replacement surgeries.

The titanium foams were suitably developed with 30 vol.% (Ti30) and 60 vol.% (Ti60) of nominal porosity by the space-holder technique using sodium chloride as the spacer. The porosity characterization showed high values of open, interconnected porosity (34.3 for Ti30 and 43.8 for Ti60), which is the required porosity for the hydrogel infiltration. In addition, the range for the porosity size between 200–600 is reported to be close to the optimal range of porosities for the osteoblastic cellular in-growth in porous Ti implants, which presents an interesting aspect when the hydrogel is infiltrated with dispersed cell growth factors.

In turn, the type-A gelatin-based hydrogel was developed and studied with 1, 2, and 3 wt.% of gelatin to determine the optimal hydrogel for the infiltration process. It was observed that the higher the concentration of the raw material, the greater the solid character. Furthermore, the system at 3% presented a more homogeneous microstructure compared to that obtained at a lower concentration. The system with 3% gelatin hydrogel was selected over the other 2 hydrogels with 1 and 2 wt.% of gelatin in terms of its solid character and greater pore size in the hydrogel network.

The kinetic behaviour of infiltration and degradation-release processes for the 3% hydrogel showed a higher infiltration rate in the Ti60 than in the Ti30 foams. The maximum infiltration value reached was 88 vol.% in the Ti30 foam after 120 min and 90.1 vol.% in the Ti60 foam after 75 min. Additionally, both processes follow an approximate Lucas-Washburn behaviour, typical for the infiltration of a fluid by capillarity in interconnected porosity. The subsequent degradation-release process showed exponential degradation mechanisms, slightly faster for Ti60 (80 min) than for Ti30 (45 min). This aspect is attributable to the greater interconnected porosity open to the surface in Ti60. Finally, the optimal biocompatibility of both gelatine-based hydrogels and Ti foams has also been corroborated.

Future studies related to this work may relate to a major biological characterization of the complete hydrogel-Ti foam system through in vitro and in vivo analyses.

## Figures and Tables

**Table 1:** Critical strain, G′, tan δ at 1 Hz (G′_1_ and tan δ_1_, respectively), viscosity (η) at 100 s^−1^ (η_100_), kinetic constant (k) and degradation mechanism (n) obtained for the hydrogels as a function of the biopolymer concentration (1, 2, and 3 wt.%).

Systems	Critical Strain (%)	G′_1_ (Pa)	tan δ_1_ (-)	η_100_ (Pa·s)	k (min^−n^)	n
1% hydrogel	0.5 ± 0.2	17.9 ± 4.1	0.26 ± 0.03	0.06 ± 0.05	51.9	0.19
2% hydrogel	20.6 ± 6.6	18.3 ± 3.7	0.19 ± 0.02	0.63 ± 0.04	17.3	0.51
3% hydrogel	14.8 ± 0.3	1983 ± 75.5	0.04 ± 0.01	3.69 ± 0.21	8.6	0.64

**Table 2:** Density and porosity characterisation of Ti30 and Ti60 foams by the Archimedes method. The theoretical density used for Ti (20 °C) = 4.5 g·cm^−3^.

Specimen	Absolute Density (g·cm^−3^)	Relative Densification (%)	Total Porosity (%)	Open Porosity (%)	Closed Porosity (%)
Ti30	2.8 ± 0.2	61.0 ± 2.7	39.0 ± 2.7	34.3 ± 2.1	4.7 ± 2.5
Ti60	1.8 ± 0.1	39.6 ± 0.1	60.4 ± 0.1	43.8 ± 3.3	16.6 ± 6.3

## Data Availability

The raw/processed data required to reproduce these findings cannot be shared at this time as the data also forms part of an ongoing study.

## References

[1] Chicardi E., Gutiérrez-González C.F., Sayagués M.J., García-Garrido C. (2018). Development of a novel TiNbTa material potentially suitable for bone replacement implants. Mater. Des..

[2] Giner M., Chicardi E., de Costa A.F., Santana L., Vázquez-Gámez M.Á., García-Garrido C., Colmenero M.A., Olmo-Montes F.J., Torres Y., Montoya-García M.J. (2021). Biocompatibility and Cellular Behavior of TiNbTa Alloy with Adapted Rigidity for the Replacement of Bone Tissue. Metals.

[3] Parithimarkalaignan S., Padmanabhan T.V. (2013). Osseointegration: An Update. J. Indian Prosthodont. Soc..

[4] Geetha M., Singh A.K., Asokamani R., Gogia A.K. (2009). Ti based biomaterials, the ultimate choice for orthopaedic implants—A review. Prog. Mater. Sci..

[5] Wong J.Y., Bronzino J.D. (2007). Biomaterials.

[6] Russo T., De Santis R., Gloria A., Barbaro K., Altigeri A., Fadeeva I., Rau J. (2020). Modification of PMMA Cements for Cranioplasty with Bioactive Glass and Copper Doped Tricalcium Phosphate Particles. Polymers.

[7] Liu X., Chen S., Tsoi J.K.H., Matinlinna J.P. (2017). Binary titanium alloys as dental implant materials—A review. Regen. Biomater..

[8] Gottlow J., Dard M., Kjellson F., Obrecht M., Sennerby L. (2012). Evaluation of a New Titanium-Zirconium Dental Implant: A Biomechanical and Histological Comparative Study in the Mini Pig. Clin. Implant Dent. Relat. Res..

[9] Li Z., Aik Khor K. (2019). Preparation and Properties of Coatings and Thin Films on Metal Implants. Encycl. Biomed. Eng..

[10] Saini M., Singh Y., Arora P., Arora V., Jain K. (2015). Implant biomaterials: A comprehensive review. World J. Clin. Cases WJCC.

[11] Ellakany P., AlGhamdi M.A., Alshehri T., Abdelrahman Z. (2022). Cytotoxicity of Commercially Pure Titanium (cpTi), Silver-Palladium (Ag-Pd), and Nickel-Chromium (Ni-Cr) Alloys Commonly Used in the Fabrication of Dental Prosthetic Restorations. Cureus.

[12] Costa B.C., Tokuhara C.K., Rocha L.A., Oliveira R.C., Lisboa-Filho P.N., Costa Pessoa J. (2019). Vanadium ionic species from degradation of Ti-6Al-4V metallic implants: In vitro cytotoxicity and speciation evaluation. Mater. Sci. Eng. C.

[13] Li Y., You Y., Li B., Song Y., Ma A., Chen B., Han W., Li C. (2019). Improved Cell Adhesion and Osseointegration on Anodic Oxidation Modified Titanium Implant Surface. J. Hard Tissue Biol..

[14] Sakka S., Coulthard P. (2011). Implant failure: Etiology and complications. Med. Oral Patol. Oral Cir. Bucal.

[15] Shi Q., Qian Z., Liu D., Liu H. (2017). Surface Modification of Dental Titanium Implant by Layer-by-Layer Electrostatic Self-Assembly. Front. Physiol..

[16] Matos G.R.M. (2021). Surface Roughness of Dental Implant and Osseointegration. J. Maxillofac. Oral Surg..

[17] Syam S., Wu C.-J., Lan W.-C., Ou K.-L., Huang B.-H., Lin Y.-Y., Saito T., Tsai H.-Y., Chuo Y.-C., Yen M.-L. (2021). The Potential of a Surface-Modified Titanium Implant with Tetrapeptide for Osseointegration Enhancement. Appl. Sci..

[18] Elias C.N., Meirelles L. (2010). Improving osseointegration of dental implants. Expert Rev. Med. Devices.

[19] Nuss K.M., Rechenberg B. (2008). von Biocompatibility Issues with Modern Implants in Bone—A Review for Clinical Orthopedics. Open Orthop. J..

[20] Abdel-Hady Gepreel M., Niinomi M. (2013). Biocompatibility of Ti-alloys for long-term implantation. J. Mech. Behav. Biomed. Mater..

[21] Wilk A., Szypulska-Koziarska D., Wiszniewska B. (2017). The toxicity of vanadium on gastrointestinal, urinary and reproductive system, and its influence on fertility and fetuses malformations. Postepy Hig. Med. Dosw..

[22] Thomas M.V., Puleo D.A. (2011). Infection, Inflammation, and Bone Regeneration: A Paradoxical Relationship. J. Dent. Res..

[23] Chocholata P., Kulda V., Babuska V. (2019). Fabrication of Scaffolds for Bone-Tissue Regeneration. Materials.

[24] Petros R.A., DeSimone J.M. (2010). Strategies in the design of nanoparticles for therapeutic applications. Nat. Rev. Drug Discov..

[25] Kang S., Hou S., Chen X., Yu D.-G., Wang L., Li X.R., Williams G. (2020). Energy-Saving Electrospinning with a Concentric Teflon-Core Rod Spinneret to Create Medicated Nanofibers. Polymers.

[26] Sharma S., Tiwari S. (2020). A review on biomacromolecular hydrogel classification and its applications. Int. J. Biol. Macromol..

[27] Ali I., Althakfi S.H., Suhail M., Locatelli M., Hsieh M.-F., Alsehli M., Hameed A.M. (2022). Advances in Polymeric Colloids for Cancer Treatment. Polymers.

[28] Albensi B.C. (2019). What Is Nuclear Factor Kappa B (NF-κB) Doing in and to the Mitochondrion?. Front. Cell Dev. Biol..

[29] Hao M., Peng X., Sun S., Ding C., Liu W. (2022). Chitosan/Sodium Alginate/Velvet Antler Blood Peptides Hydrogel Promoted Wound Healing by Regulating PI3K/AKT/mTOR and SIRT1/NF-κB Pathways. Front. Pharmacol..

[30] Hao T., Qian M., Zhang Y., Liu Q., Midgley A.C., Liu Y., Che Y., Hou J., Zhao Q. (2022). An Injectable Dual-Function Hydrogel Protects Against Myocardial Ischemia/Reperfusion Injury by Modulating ROS/NO Disequilibrium. Adv. Sci..

[31] Zhang C., Cheng Z., Zhou Y., Yu Z., Mai H., Xu C., Zhang J., Wang J. (2022). The novel hyaluronic acid granular hydrogel attenuates osteoarthritis progression by inhibiting the TLR-2/ NF-κB signaling pathway through suppressing cellular senescence. Bioeng. Transl. Med..

[32] Chafran L., Carfagno A., Altalhi A., Bishop B. (2022). Green Hydrogel Synthesis: Emphasis on Proteomics and Polymer Particle-Protein Interaction. Polymers.

[33] Sola D., Cases R. (2020). High-Repetition-Rate Femtosecond Laser Processing of Acrylic Intra-Ocular Lenses. Polymers.

[34] Ahmed E.M. (2015). Hydrogel: Preparation, characterization, and applications: A review. J. Adv. Res..

[35] Augst A.D., Kong H.J., Mooney D.J. (2006). Alginate Hydrogels as Biomaterials. Macromol. Biosci..

[36] Zhao H.Y., Wu J., Zhu J.J., Xiao Z.C., He C.C., Shi H.X., Li X.K., Yang S.L., Xiao J. (2015). Research Advances in Tissue Engineering Materials for Sustained Release of Growth Factors. Biomed Res. Int..

[37] Lv H., Guo S., Zhang G., He W., Wu Y., Yu D.-G. (2021). Electrospun Structural Hybrids of Acyclovir-Polyacrylonitrile at Acyclovir for Modifying Drug Release. Polymers.

[38] Yaszemski M.J., Payne R.G., Hayes W.C., Langer R.S., Aufdemorte T.B., Mikos A.G. (2007). The Ingrowth of New Bone Tissue and Initial Mechanical Properties of a Degrading Polymeric Composite Scaffold. Tissue Eng..

[39] Ahmady A., Abu Samah N.H. (2021). A review: Gelatine as a bioadhesive material for medical and pharmaceutical applications. Int. J. Pharm..

[40] Perez-Puyana V., Jiménez-Rosado M., Romero A., Guerrero A. (2020). Fabrication and characterization of hydrogels based on gelatinised collagen with potential application in tissue engineering. Polymers.

[41] Essawy H.A., Ghazy M.B.M., El-Hai F.A., Mohamed M.F. (2016). Superabsorbent hydrogels via graft polymerization of acrylic acid from chitosan-cellulose hybrid and their potential in controlled release of soil nutrients. Int. J. Biol. Macromol..

[42] Korsmeyer R.W., Gurny R., Doelker E., Buri P., Peppas N.A. (1983). Mechanisms of solute release from porous hydrophilic polymers. Int. J. Pharm..

[43] Pranantyo D., Kang E.-T., Chan-Park M.B. (2021). Smart nanomicelles with bacterial infection-responsive disassembly for selective antimicrobial applications. Biomater. Sci..

[44] Strober W. (2015). Trypan Blue Exclusion Test of Cell Viability. Curr. Protoc. Immunol..

[45] Rodriguez-Contreras A., Punset M., Calero J.A., Gil F.J., Ruperez E., Manero J.M. (2021). Powder metallurgy with space holder for porous titanium implants: A review. J. Mater. Sci. Technol..

[46] Sánchez-Cid P., Jiménez-Rosado M., Alonso-González M., Romero A., Perez-Puyana V. (2021). Applied Rheology as Tool for the Assessment of Chitosan Hydrogels for Regenerative Medicine. Polymers.

[47] Bartnikowski M., Wellard R.M., Woodruff M., Klein T. (2015). Tailoring Hydrogel Viscoelasticity with Physical and Chemical Crosslinking. Polymers.

[48] Sánchez-Cid P., Jiménez-Rosado M., Perez-Puyana V., Guerrero A., Romero A. (2021). Rheological and Microstructural Evaluation of Collagen-Based Scaffolds Crosslinked with Fructose. Polymers.

[49] George B., Bhatia N., Kumar A., SK S., Vadakkadath Meethal K., TM S., TV S. (2022). Bioinspired gelatin based sticky hydrogel for diverse surfaces in burn wound care. Sci. Rep..

[50] Ahmadian E., Eftekhari A., Kavetskyy T., Khosroushahi A.Y., Turksoy V.A., Khalilov R. (2020). Effects of quercetin loaded nanostructured lipid carriers on the paraquat-induced toxicity in human lymphocytes. Pestic. Biochem. Physiol..

[51] Kanokpanont S., Damrongsakkul S., Ratanavaraporn J., Aramwit P. (2012). An innovative bi-layered wound dressing made of silk and gelatin for accelerated wound healing. Int. J. Pharm..

[52] Conrad B., Hayashi C., Yang F. (2021). Gelatin-Based Microribbon Hydrogels Guided Mesenchymal Stem Cells to Undergo Endochondral Ossification In Vivo with Bone-Mimicking Mechanical Strength. Regen. Eng. Transl. Med..

[53] Cao C., Yang N., Zhao Y., Yang D., Hu Y., Yang D., Song X., Wang W., Dong X. (2021). Biodegradable hydrogel with thermo-response and hemostatic effect for photothermal enhanced anti-infective therapy. Nano Today.

[54] Chang W.-C., Tai A.-Z., Tsai N.-Y., Li Y.-C.E. (2021). An Injectable Hybrid Gelatin Methacryloyl (GelMA)/Phenyl Isothiocyanate-Modified Gelatin (Gel-Phe) Bioadhesive for Oral/Dental Hemostasis Applications. Polymers.

[55] Torres-Sanchez C., Norrito M., Almushref F.R., Conway P.P. (2021). The impact of multimodal pore size considered independently from porosity on mechanical performance and osteogenic behaviour of titanium scaffolds. Mater. Sci. Eng. C.

[56] Zhang L.C., Chen L.Y. (2019). A Review on Biomedical Titanium Alloys: Recent Progress and Prospect. Adv. Eng. Mater..

[57] Morgan E.F., Unnikrisnan G.U., Hussein A.I. (2018). Bone Mechanical Properties in Healthy and Diseased States. Annu. Rev. Biomed. Eng..

[58] Lloreda-Jurado P.J., Chicardi E., Paúl A., Sepúlveda R. (2021). Effect of processing parameters on the properties of freeze-cast Ni wick with gradient porosity. Mater. Des..

